# Case report: Emergency management of adjacent arterial injury during inferior vena cava angioplasty in a patient with Budd-Chiari syndrome

**DOI:** 10.3389/fmed.2026.1846905

**Published:** 2026-06-16

**Authors:** Yue Luo, Wei Duan, Wei Li, Weibing Leng

**Affiliations:** 1 Interventional Radiology Center, West China Hospital of Sichuan University/West China School of Nursing, Sichuan University, Chengdu, China; 2 Intervention Center, West China Hospital, Sichuan University, Chengdu, China

**Keywords:** arterial puncture, Budd-Chiari syndrome, case report, endovascular therapy, inferior vena cava angioplasty

## Abstract

Budd-Chiari syndrome (BCS) is a clinical syndrome caused by stenosis or occlusion of the hepatic veins and/or the inferior vena cava (IVC) near their orifices. Endovascular therapy is a common therapeutic approach. However, the puncture procedure during angioplasty may damage adjacent arteries, compromising patient safety and affecting clinical outcomes. This report describes a case of a patient with BCS who suffered an inadvertent injury to the origin of the right common carotid artery (CCA) during IVC angioplasty under angiographic guidance. Through multidisciplinary consultation and emergency intervention, three covered stents were precisely placed at the bleeding site, achieving successful hemostasis. This case report aims to provide a guidance for the early clinical recognition and management of such intraprocedural complications.

## Introduction

Budd-Chiari syndrome (BCS) is a clinical syndrome caused by stenosis or occlusion of the hepatic veins and/or the inferior vena cava (IVC) near their orifices (1, 2). The reported annual incidence of BCS ranges from 0.17 to 4.1 cases per 100,000 persons, with a prevalence of 1.4 to 7.69 per 100,000. Recent studies indicate a higher incidence in European populations than in Asian populations (3). Endovascular therapy is the first-line treatment for BCS; effective recanalization of occluded vessels helps alleviate or eliminate patient symptoms (4). Complications of endovascular therapy are mostly related to local injury from intraoperative puncture, balloon dilation, and stent placement. Common complications include cardiac tamponade, IVC rupture, and hepatic capsule rupture with bleeding, all of which affect patient safety during surgery and disease prognosis (5, 6). Currently, there are few worldwide reports of intraoperative puncture injuries involving complex arterial anatomy, and relevant emergency management experience is lacking.

## Case presentation

On November 22, 2025, a 62-year-old male patient was admitted to the Department of Gastroenterology at a tertiary hospital in China. Five months prior, the patient developed nausea without obvious cause, accompanied by mild skin yellowing, pruritus, minor gingival bleeding, intermittent bilateral lower extremity edema, and fatigue. The diagnoses were primary hepatocellular carcinoma T4N0M0 stage IIB, cirrhosis with esophageal and gastric varices, Budd-Chiari syndrome, chronic renal insufficiency, etc. Four months prior, the patient underwent transcatheter arterial chemoembolization (TACE) + hepatic artery angiography + celiac artery angiography under local anesthesia and was started on “lenvatinib 2 capsules once daily. The patient underwent hepatic vein CT venography + energy imaging showed the hepatic segment of the IVC was locally markedly slender and poorly visualized. Two months prior, the patient was hospitalized at the same institution for further workup and underwent TACE and IVC angioplasty. Twenty-two days prior, an upper abdominal MRI with plain, contrast-enhanced thin-slice, and specialized imaging showed possible hepatocellular carcinoma with intrahepatic metastasis; the lesions had decreased in size compared to previous, the hepatic segment of the IVC was partially poorly visualized. The patient was now admitted for evaluation for IVC angioplasty.

On admission, the patient was alert and in fair general condition, without significant complaints. MRI of the upper abdomen (plain, angiographic, and contrast-enhanced thin-slice) showed BCS, cirrhosis, splenomegaly, and a small amount of ascites were present. Laboratory findings: total bilirubin 29.5 μmol/L, direct bilirubin 12.8 μmol/L, *γ*-glutamyl transferase 80 U/L, total protein 50.3 g/L, albumin 29.0 g/L, creatinine 140.0 μmol/L, uric acid 438 μmol/L, mean corpuscular volume 77.5 fL, mean corpuscular hemoglobin 24.5 pg., RBC distribution width CV 16.9%, platelet count 60 × 10^9^/L, white blood cell count 2.45 × 10^9^/L, eosinophil percentage 11.6%, absolute neutrophil count 1.13 × 10^9^/L, absolute lymphocyte count 0.79 × 10^9^/*L. alpha*-fetoprotein 130.00 ng/mL; abnormal prothrombin 183.00 mAU/mL. Past medical history was unremarkable, with no history of infectious diseases or trauma. He had smoked for 30 years but quit >10 years ago; no alcohol use; no family or genetic history.

Physical examination on admission: T 36.3 °C, P 101 beats/min, R 20 breaths/min, BP 118/74 mmHg, HR 101 beats/min, height 166 cm, weight 59 kg. Normal abdominal contour, soft abdomen, no tenderness or rebound tenderness, no palpable abdominal mass, liver and spleen not palpable below the costal margin, kidneys not palpable.

## Procedure and outcome

**Figure 1:**
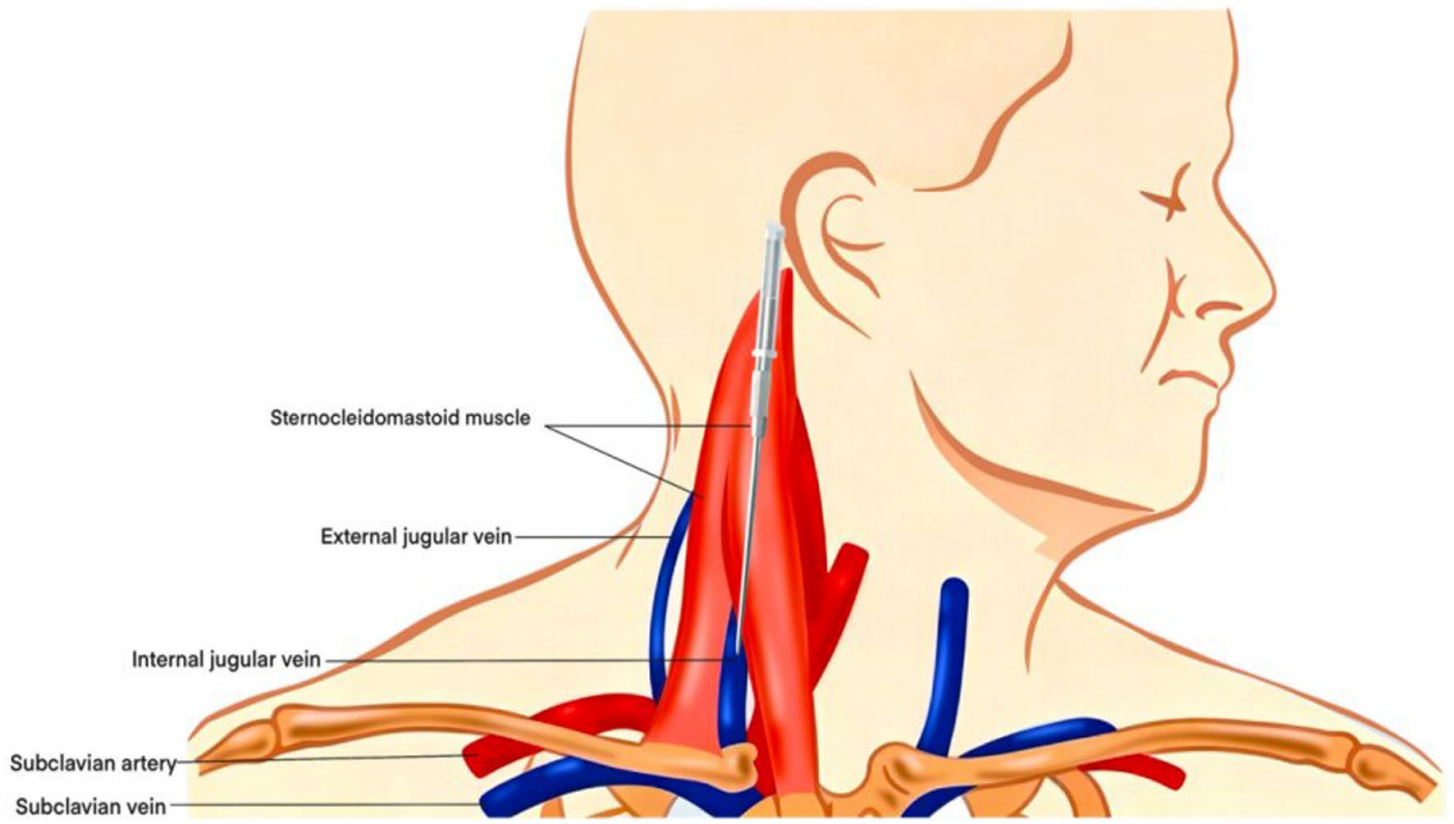
Schematic diagram of internal jugular vein puncture site.

**Figure 2:**
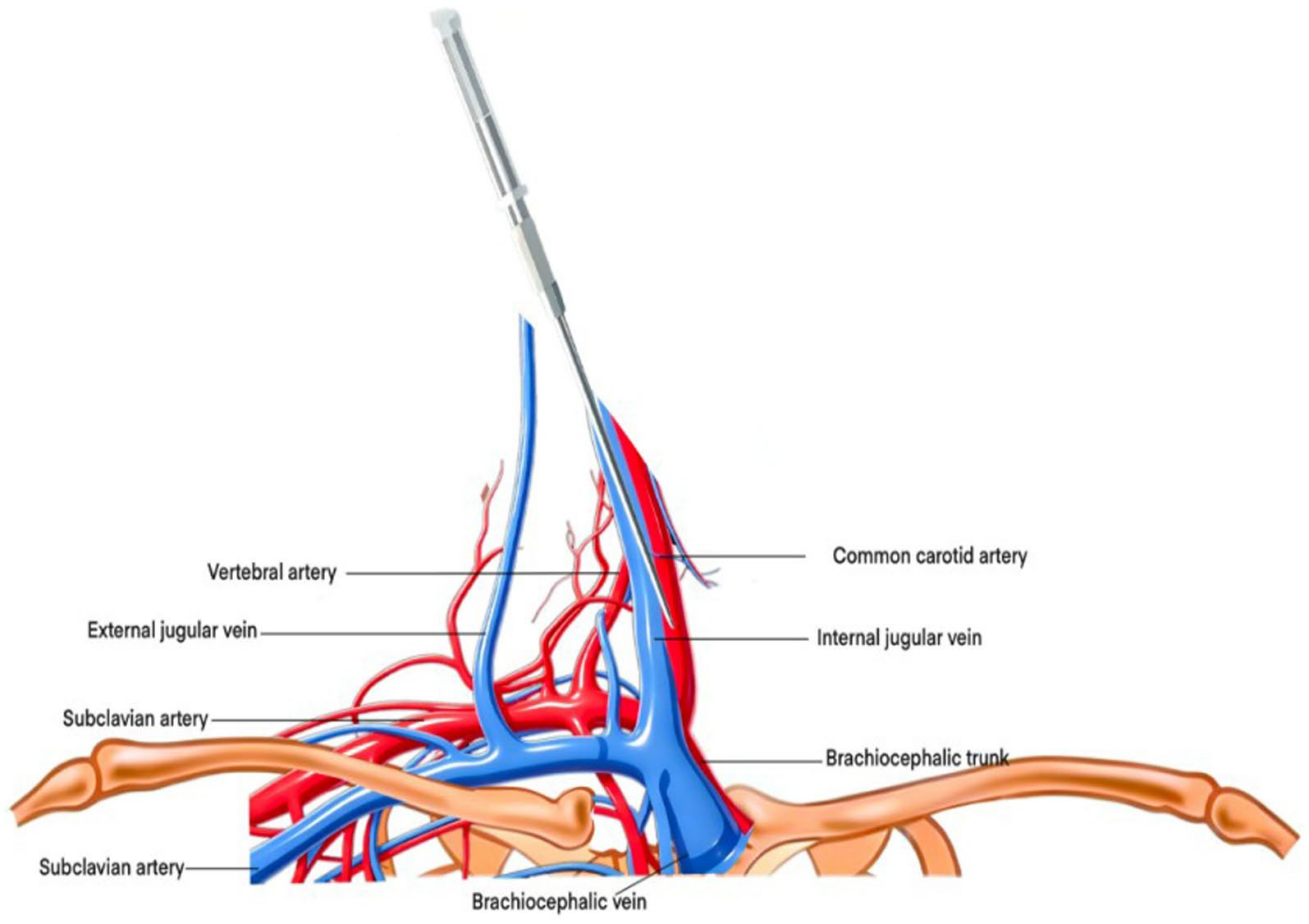
Accidental injury to the origin of the common carotid artery during internal jugular vein puncture.

The patient was scheduled for IVC angioplasty in the interventional radiology suite on November 25, 2025. After local anesthesia (2% lidocaine hydrochloride injection, subcutaneous, 5 mL), a interventional radiologist punctured the right internal jugular vein and advanced a catheter into the IVC. Angiography showed no enhancement of the retrohepatic segment of the IVC, indicating occlusion, along with multiple collateral circulations. A pressure gradient of 10 mmHg was measured between the distal IVC and the right atrium. Balloon dilation was planned. However, during preparation to exchange the outer sheath, significant oozing was observed at the neck puncture site, along with a subcutaneous hematoma, raising suspicion of adjacent arterial injury and bleeding (Figures 1, 2).

Due to the unique anatomical location, a multidisciplinary team (MDT) was convened, comprising cardiac surgery, vascular surgery, thoracic surgery, head and neck surgery, and neurology. The team discussed treatment strategies while preparing for emergency open surgery. After puncturing the femoral artery with a 5F short sheath, a 0.035-inch guidewire with a 100 cm multi-purpose catheter was used coaxially to perform angiography of the aortic arch, which revealed no bleeding point. Subsequent angiography of the CCA showed that bleeding originated from a rupture at the origin of the right CCA, assessed as a puncture injury. Neurologic examination revealed no significant abnormalities. After MDT discussion, the procedure was handed over to a neurointerventionalist as the primary operator, with gastroenterology and vascular surgery physicians assisting.

**Figure 3:**
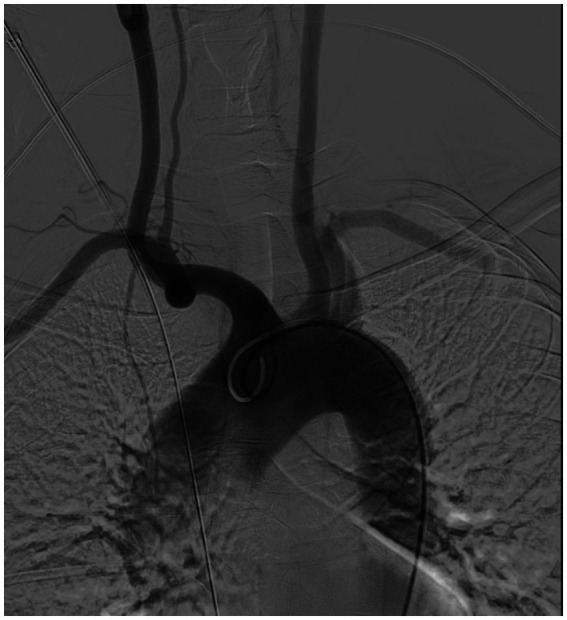
Aortic arch angiography showing no bleeding point.

**Figure 4:**
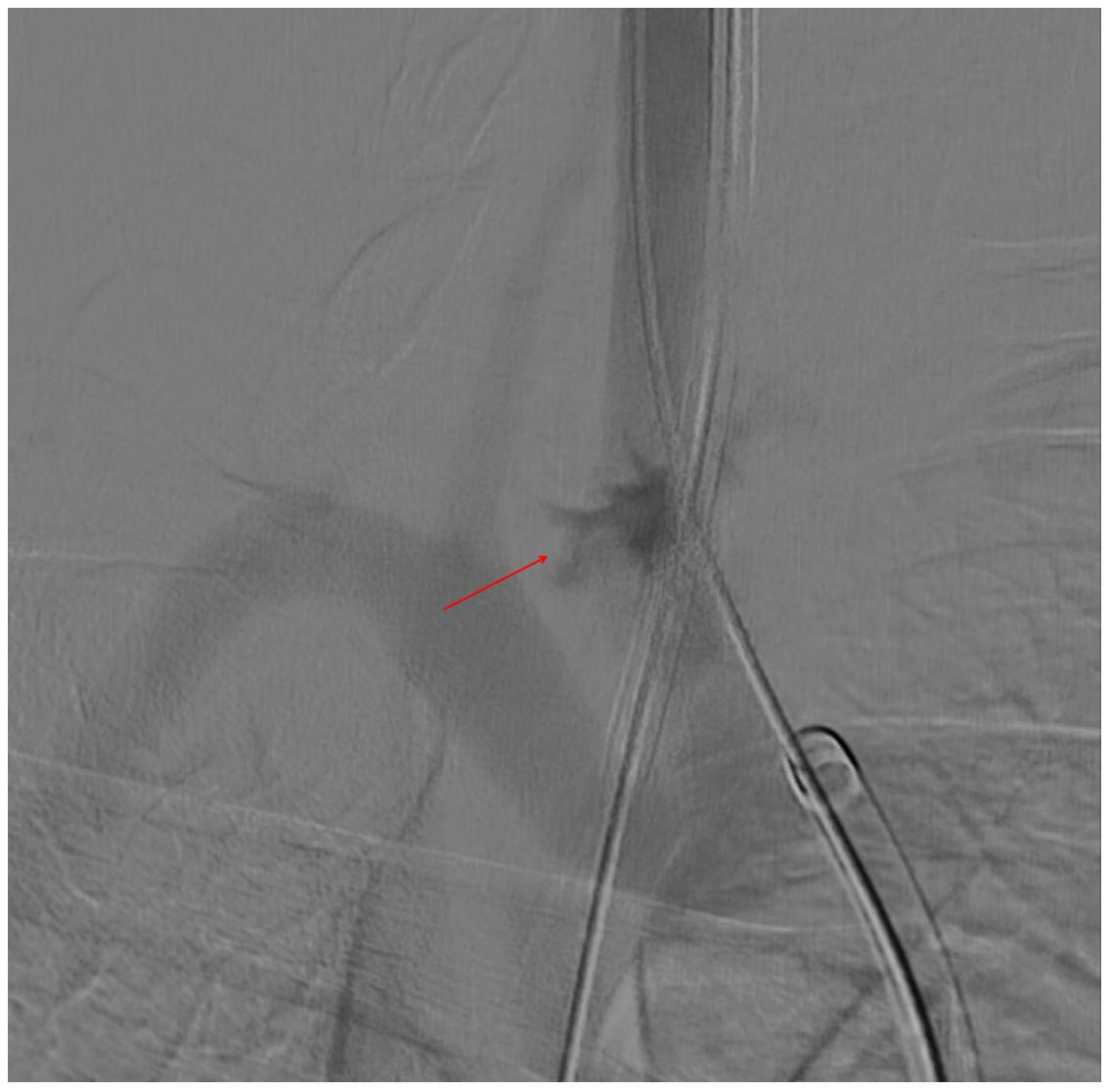
Common carotid artery angiography revealing the bleeding point.

**Figure 5:**
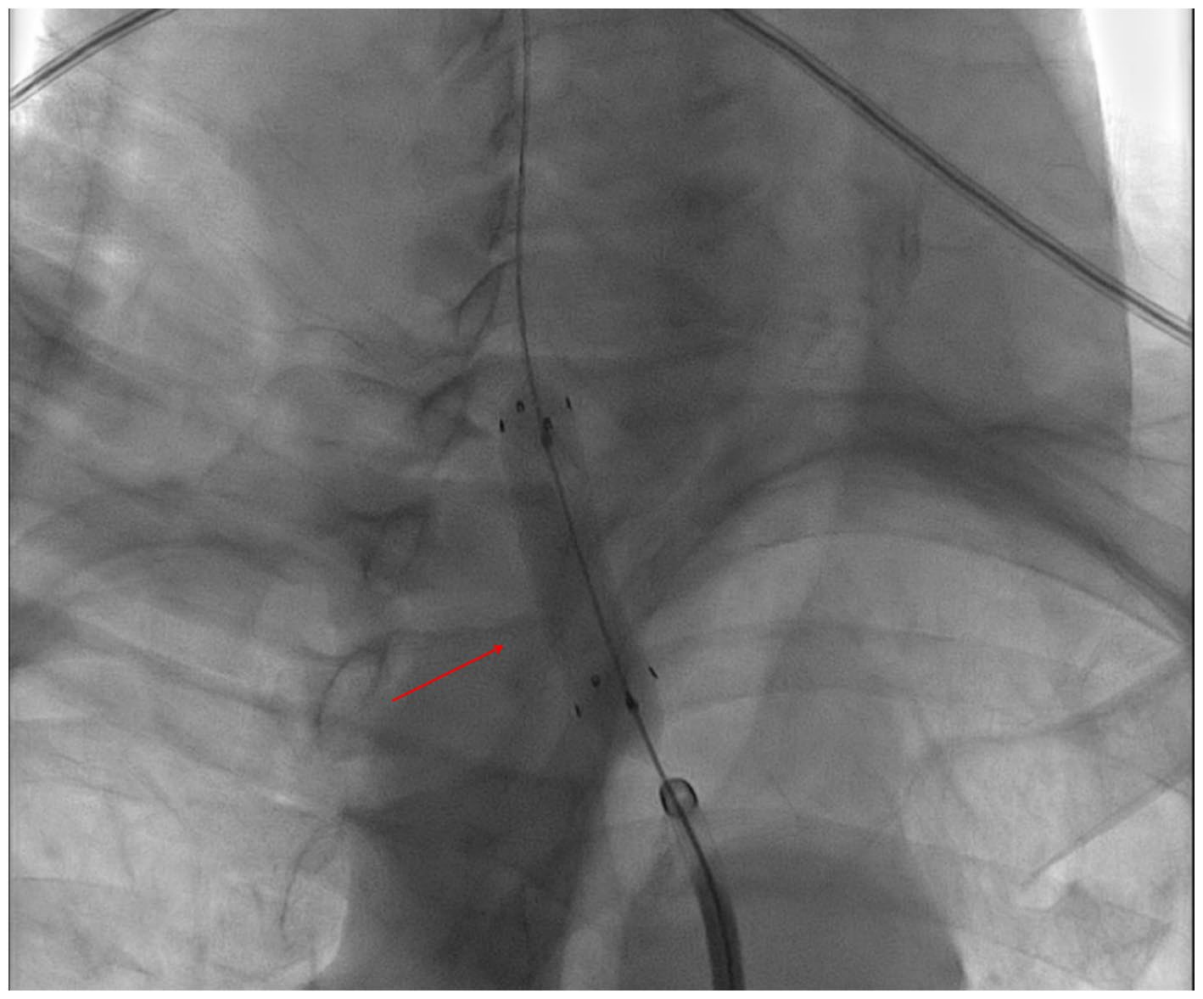
Placement of covered stent in the common carotid artery with balloon dilation for apposition.

**Figure 6:**
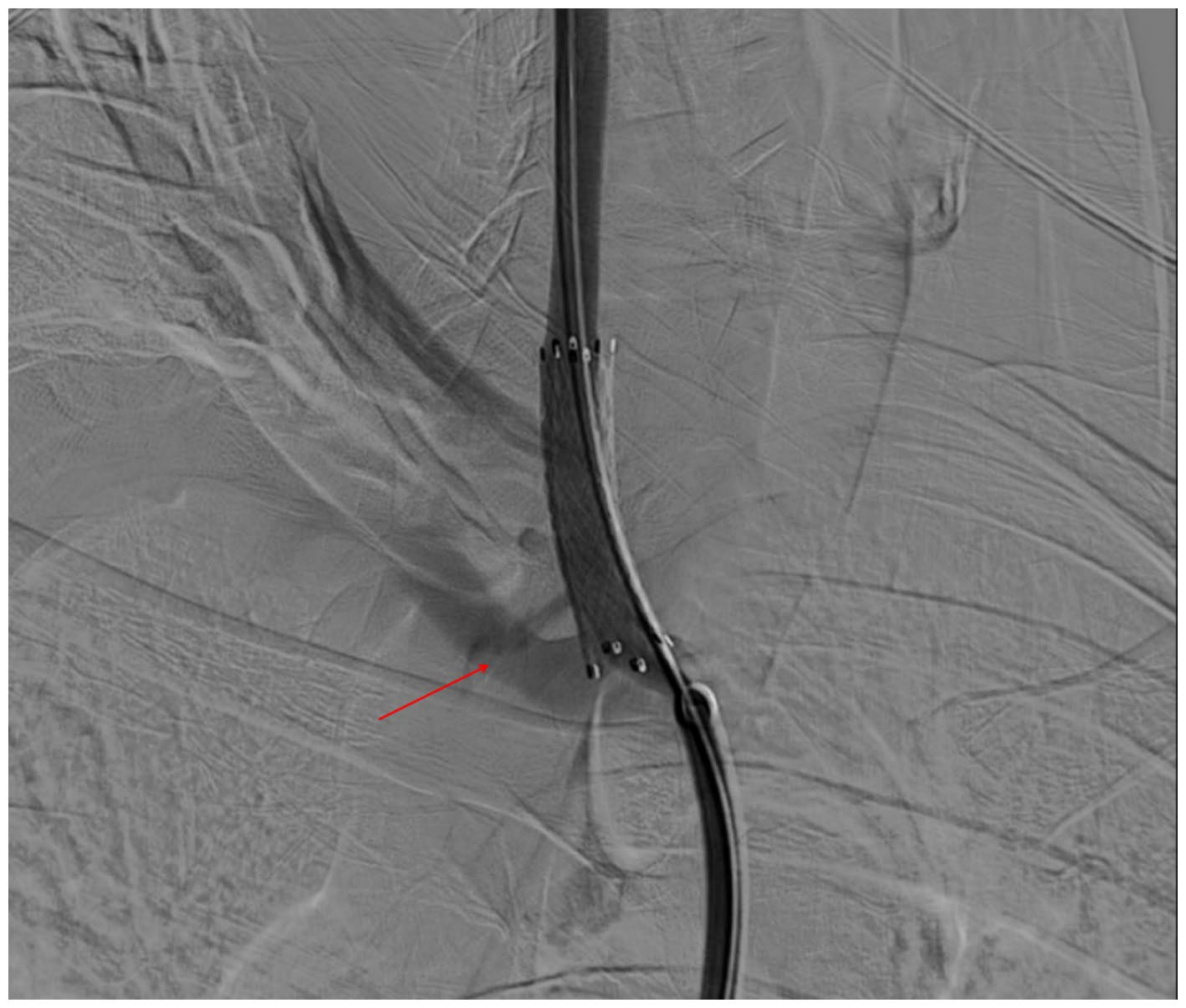
Persistent minor bleeding after placement of two covered stents.

**Figure 7:**
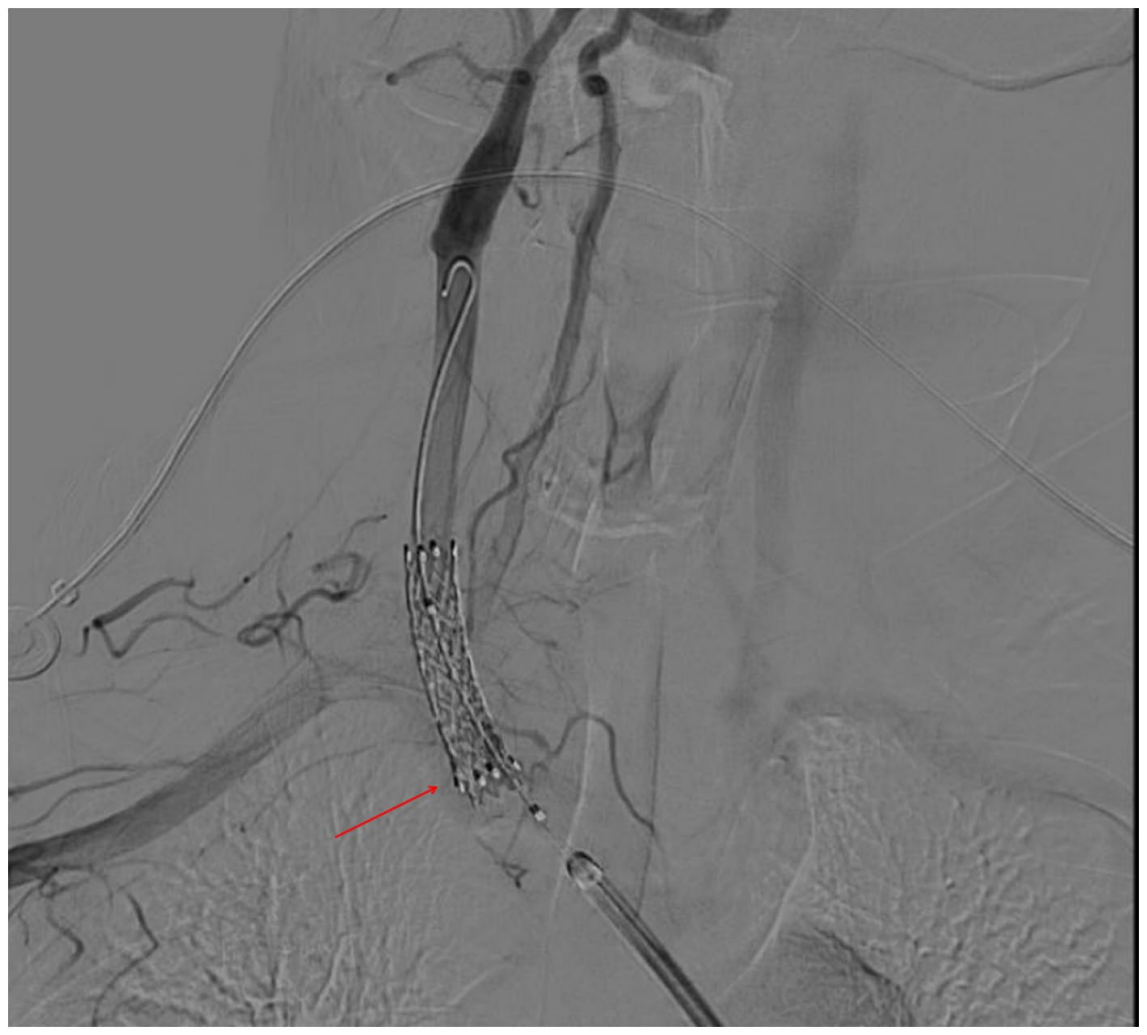
Intraoperative placement of three covered stents.

**Figure 8:**
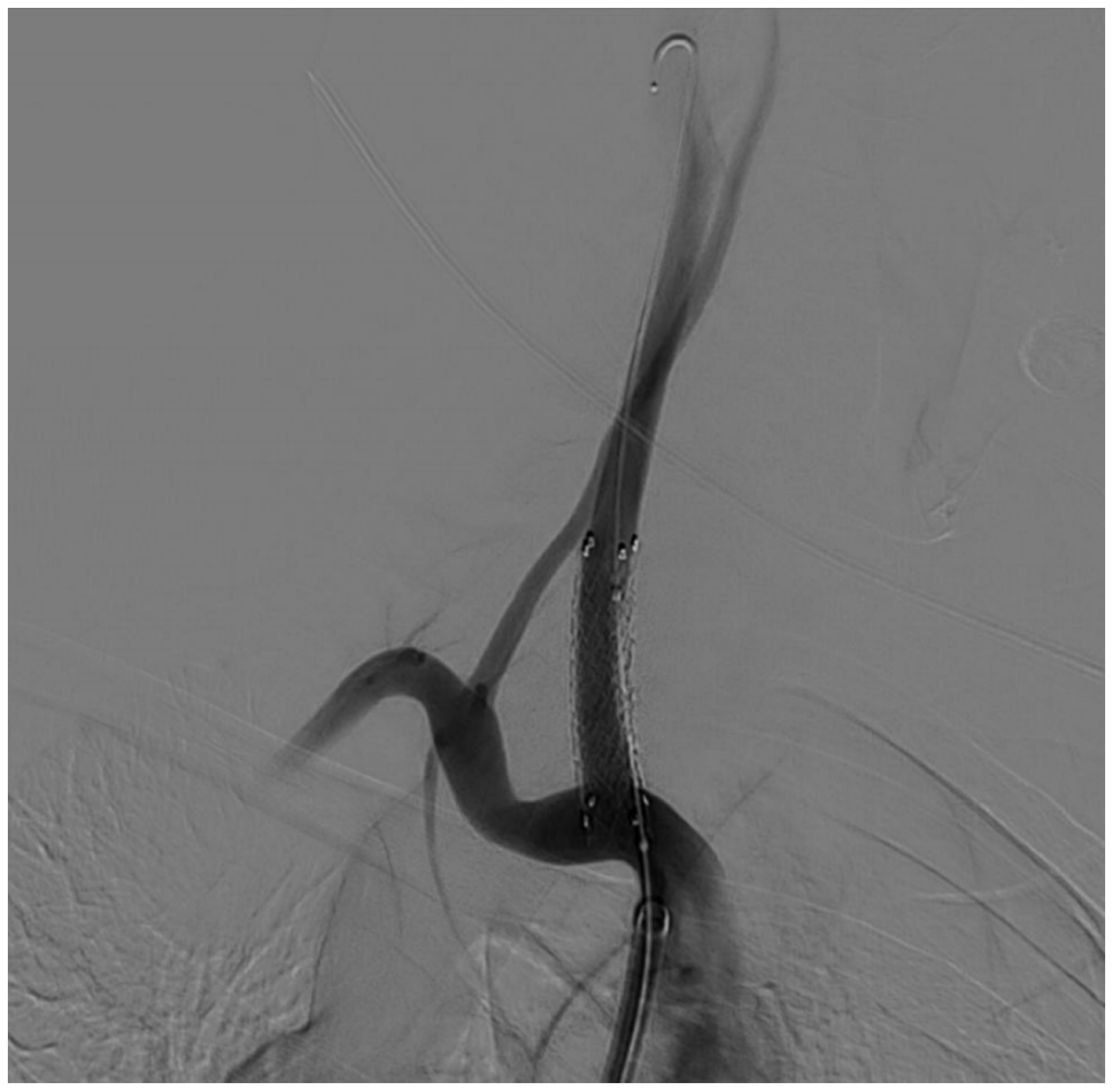
Post-procedure image showing resolution of bleeding at the origin of the common carotid artery; hemostasis achieved.

A 10F vascular long sheath was exchanged. A 0.035-inch guidewire with a 125 cm multi-purpose catheter was used coaxially to advance the 10F long sheath into the brachiocephalic trunk. An Amplatz super-stiff guidewire, with its tip looped and placed at the distal end of the right CCA, served as the working wire. Multi-angle angiography confirmed bleeding from the injured origin of the right CCA. One 8 × 40 mm Fluency Plus covered stent was accurately implanted at the bleeding site of the right CCA. Repeat angiography showed persistent active bleeding. A second overlapping covered stent (9 × 38 mm Lifestream) was implanted within the first stent. Repeat angiography still showed a small amount of bleeding. A third covered stent (10 × 38 mm Lifestream) was then implanted in the proximal segment of the stent column (total of three stents). Final angiography showed good stent position and wall apposition, with no active bleeding. Post-procedure neurological examination revealed no new significant abnormalities. The right femoral artery puncture site was sutured and compressed with bandaging, and the procedure was terminated (Figures 3–8).

Upon detection of bleeding, the procedure was immediately halted. The short sheath in the internal jugular vein was left in place to prevent secondary injury and to help reduce bleeding. Blood typing and crossmatching were performed immediately, and an emergency transfusion protocol was prepared. Multiple intraoperative blood gas analyses showed hemoglobin fluctuating between 92–100 g/L. The patient had impaired venous return from the lower extremities, abdomen, and pelvis, with ascites and lower extremity edema. Aggressive fluid resuscitation could increase circulatory burden and worsen liver congestion. Additionally, preoperative liver and kidney function tests showed chronic renal insufficiency and severely impaired hepatic synthetic function. Intraoperative blood pressure remained within normal range (systolic 90–140 mmHg); therefore, a restrictive fluid strategy was adopted, and a total of 400 mL of lactated Ringer’s solution was infused.

The patient had not received antiplatelet therapy preoperatively. Tirofiban was calculated at 0.1 μg/kg/min (7). For a patient weighing 59 kg, the dose of tirofiban sodium chloride injection was 7 mL/h (2.5 mg intravenously via micro-pump). After CT scan confirmed no further bleeding, the patient was switched to dual antiplatelet therapy with enteric-coated aspirin (100 mg/day) and clopidogrel bisulfate (75 mg/day) orally for 6 months.

Postoperatively, the patient returned to the ward without respiratory distress, palpitations, chest tightness, chest pain, or dizziness. A subcutaneous hematoma approximately 5 cm in diameter was present near the internal jugular vein puncture site. Manual compression was applied for 2 h postoperatively, supplemented with ice packs or magnesium sulfate wet compresses. On postoperative day 2, the patient reported pain and discomfort in the neck and chest, and right scleral congestion was observed. On postoperative day 4, the pain improved, and the neck hematoma had decreased. At the outpatient follow-up on postoperative day 14, the patient’s neck pain had disappeared and the neck swelling had completely resolved. The patient’s neck pain had disappeared and the neck swelling had completely resolved.

**Figure 9:**
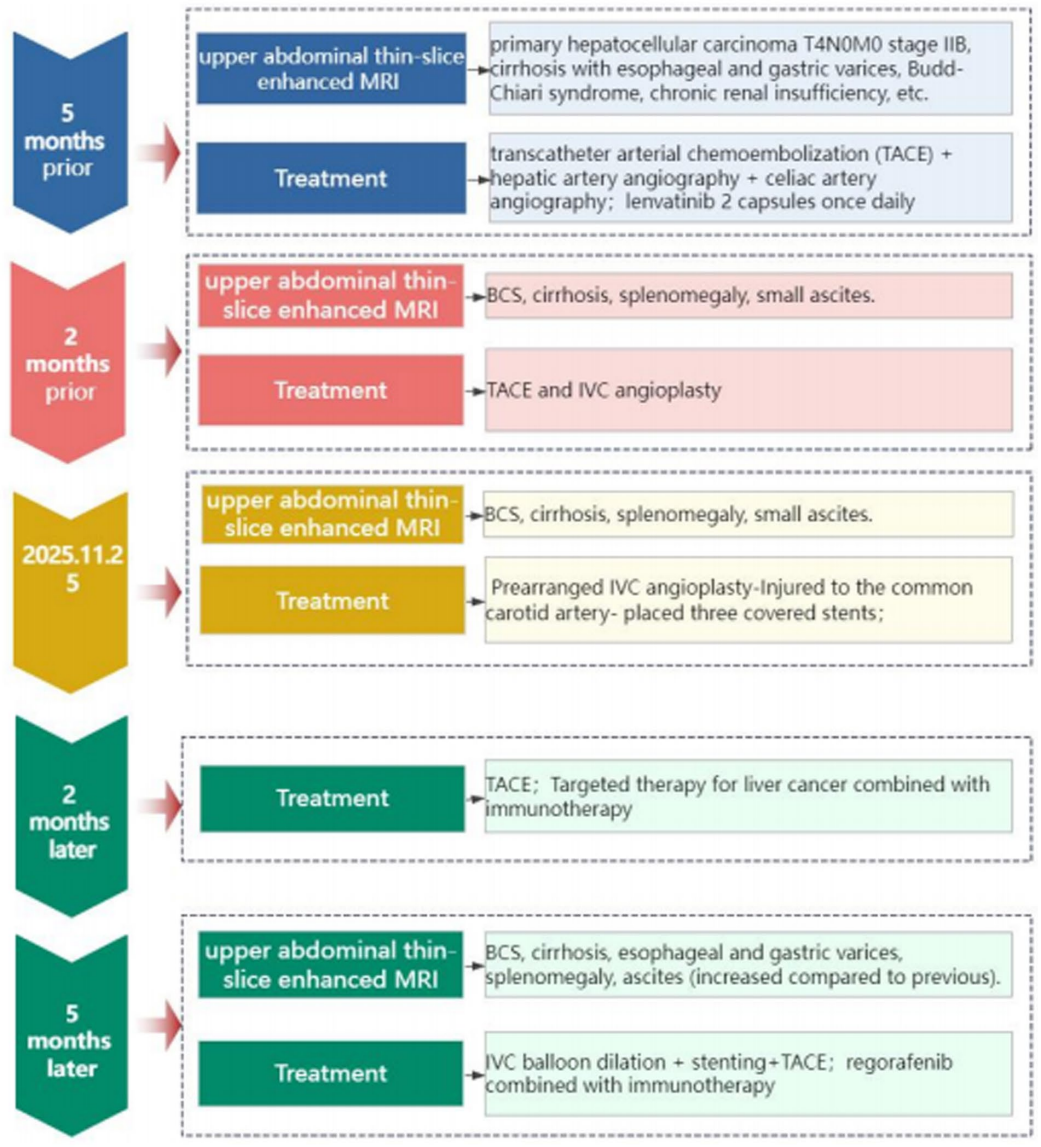
of disease progression and treatment for patients. MRI, Magnetic resonance imaging; TACE, transcatheter arterial chemoembolization; IVC, inferior vena cava; BCS, Budd-Chiari syndrome.

The patient was discharged 5 days postoperatively. Five months later, he was readmitted for IVC angioplasty (balloon dilation and stent placement) combined with TACE. The perioperative course was uneventful, and the patient was discharged 3 days afterward. The progression of the patient’s disease and treatment modalities are shown in Figure 9, and changes in liver function test results are presented in Table 1.

**Table 1:** Changes in the patient’s liver and kidney function test results.

Time point	Key laboratory values
Two months prior	Total bilirubin 20.3 μmol/L; direct bilirubin 7.4 μmol/L; aspartate aminotransferase 46 U/L; gamma-glutamyl transferase 9 U/L; total protein 60.4 g/L; albumin 35.2 g/L; creatinine 127.0 μmol/L.
Preoperatively	Total bilirubin 29.5 μmol/L; direct bilirubin 12.8 μmol/L; aspartate aminotransferase 33 U/L; gamma-glutamyl transferase 80 U/L; total protein 50.3 g/L; albumin 29.0 g/L; creatinine 140.0 μmol/L.
Postoperatively	Total bilirubin 25.2 μmol/L; direct bilirubin 12.0 μmol/L; aspartate aminotransferase 40 U/L; gamma-glutamyl transferase 72 U/L; total protein 44.8 g/L; albumin 27.2 g/L; creatinine 146.0 μmol/L.
Two months later	Total bilirubin 18.2 μmol/L; direct bilirubin 9.6 μmol/L; aspartate aminotransferase 74 U/L; gamma-glutamyl transferase 130 U/L; total protein 50.3 g/L; albumin 30.2 g/L; creatinine 149.0 μmol/L.
Five months later	Total bilirubin 31.6 μmol/L; direct bilirubin 12.9 μmol/L; aspartate aminotransferase 49 U/L; gamma-glutamyl transferase 126 U/L; total protein 55.1 g/L; albumin 31.8 g/L; creatinine 159.0 μmol/L.

### Follow-up

The patient was scheduled for a structured 6-month follow-up. Five follow-up visits have been completed over 5 months. Patient assessment showed complete resolution of neck pain and disappearance of the subcutaneous hematoma. The patient fully adhered to anticoagulant medication and reported no melena, gingival bleeding, or ecchymosis. No adverse events or unexpected complications occurred during follow-up. Five months later, the systemic therapy for hepatocellular carcinoma was adjusted to regorafenib combined with immunotherapy. Repeat blood count, liver function, quantitative AFP, abnormal prothrombin, and contrast-enhanced upper abdominal MRI are planned after 2 months, and follow-up will continue.

## Discussion

Despite advances in endovascular and surgical techniques, management of intraprocedural vascular injury remains a challenging clinical problem. Traditional treatment strategies for open iatrogenic vascular injury typically include compression, packing, surgical suture repair, vessel sacrifice, and bypass grafting (8). Iatrogenic carotid artery rupture has been reported in 3–5% of patients undergoing major head and neck surgery. Carotid artery injury or rupture is uncommon in clinical practice, the mortality rate can range from 17 to 40%, and the reported incidence of severe central nervous system complications among survivors is 40–80% (9). Carotid artery injuries can be caused by trauma, cancer or cancer treatment, and spontaneous dissection, among other factors, interventional therapy offers an alternative to traditional surgical methods for treating carotid artery injuries (10).

The right common carotid artery originates from the bifurcation of the brachiocephalic trunk behind the sternoclavicular joint. It lies anterior to the scalene and longus colli muscles and terminates at the level of the thyroid cartilage, where it divides into the internal and external carotid arteries. The internal jugular vein is usually located adjacent and slightly to the right of the common carotid artery, but anatomical variations in their spatial relationship may exist. In clinical practice, puncture of the internal jugular vein for various reasons may inadvertently injure the adjacent common carotid artery, resulting in local hematoma (11).

Endovascular therapy, particularly covered stent placement, is a promising method for treating intraprocedural arterial injury. Covered stent or stent-graft placement represents a newer endovascular concept focused on repairing the arterial wall defect, distinct from endovascular embolization or shunt therapy (12). Covered stents can be considered for treating common carotid artery injuries, including traumatic, iatrogenic, and tumor erosion. Covered stents feature a membrane on the inner, lumenal, or both sides, providing a reconstructive treatment option without vessel sacrifice, enabling immediate bleeding control (13). Placement is relatively quick and convenient, especially suitable for emergency situations. However, covered stents have drawbacks: covering branch ostia may risk branch artery occlusion; their stiffer structure, larger profile, and reduced trackability may cause secondary injury to the parent artery; and the subsequent increased risk of in-stent thrombosis, stenosis, or occlusion may limit their use (14).

The occurrence of thrombosis and in-stent stenosis after covered stent placement is influenced by multiple factors, including clinical factors, vessel diameter, vessel tortuosity, and hemodynamics (15, 16). Dual antiplatelet therapy (DAPT) is standard after carotid artery stenting, but the optimal regimen and duration of DAPT remain undetermined. For emergency internal carotid stenting, a glycoprotein IIb/IIIa receptor antagonist (e.g., tirofiban) may be used. If postoperative CT confirms no increased bleeding, oral DAPT can be used (16). After stenting, short-term (1–3 months) and subsequent annual clinical follow-up is generally recommended, with timely adjustment of antiplatelet medication strategies based on patient symptoms and follow-up imaging results.

## Limitations

Several limitations must be explicitly noted. The injured vessel in this study was the origin of the common carotid artery; the emergency management experience described may not be applicable to patients with intracranial or other peripheral arterial injuries, who should be managed on a case-by-case basis. The patient in this case had relatively modest bleeding and intraoperative blood pressure could be maintained within the normal range; therefore, this strategy may not be fully applicable to patients with massive bleeding leading to hemorrhagic shock or significant neurological deficits. Furthermore, there is a lack of follow-up laboratory and imaging reports after IVC balloon dilation combined with stenting, which requires continued follow-up in the future.

## Conclusion

This report details the emergency management of an adjacent arterial puncture injury during IVC angioplasty in a patient with Budd-Chiari syndrome. The key contributions include: (1) after urgent identification of bleeding, multidisciplinary assessment (cardiac surgery, vascular surgery, thoracic surgery, head and neck surgery, and neurology) led to the decision for covered stent placement, demonstrating feasibility and effectiveness; (2) after bleeding was identified, unnecessary manipulations were minimized to prevent further bleeding extension; (3) based on the patient’s bleeding status, blood volume was maintained with appropriate intraoperative blood pressure control and fluid resuscitation strategy; (4) postoperative anticoagulation and follow-up were essential.

## Data Availability

The original contributions presented in the study are included in the article/supplementary material, further inquiries can be directed to the corresponding author.
